# Differential PD-1/LAG-3 expression and immune phenotypes in metastatic sites of breast cancer

**DOI:** 10.1186/s13058-020-01380-w

**Published:** 2021-01-07

**Authors:** Bettina Sobottka, Holger Moch, Zsuzsanna Varga

**Affiliations:** Department of Pathology and Molecular Pathology, University and University Hospital Zurich, Schmelzbergstrasse 12, CH-8091 Zurich, Switzerland

**Keywords:** Breast cancer, Metastasis, Tumor immunology, Immune checkpoint receptors, Immune phenotype

## Abstract

**Background:**

A dual blockade against the novel immune checkpoint inhibitor lymphocyte activation gene-3 (LAG-3) and programmed cell death protein-1 (PD-1) is currently considered in advanced breast cancer. Nevertheless, PD-1 or LAG-3 expression within distant metastatic breast cancer tissue remains understudied.

**Methods:**

To address this knowledge gap, we investigated the PD-1 and LAG-3 expression in combination with the CD8-based immune phenotype in intrapatient matched primary tumor distant metastases, representing 95 breast cancer patients with metastases occurring at four different anatomical locations. The immune phenotype was categorized into 2 categories: inflamed corresponding to the clinical category “hot” and exhausted or desert consistent with clinically “cold” tumors.

**Results:**

Metastases of “cold” primary tumors always remained “cold” at their matched metastatic site. Expression of PD-1/LAG-3 was associated with a “hot” immune phenotype in both the primary tumors and metastases. We could not observe any association between the immune phenotype and the breast cancer molecular subtype. Brain and soft tissue metastases were more commonly inflamed with signs of exhaustion than other anatomical sites of metastases. Taken together, (i) the immune phenotype varied between sites of distant metastases, and (ii) PD-1^+^/LAG-3^+^ was strongly associated with a “hot” immune phenotype and (iii) was most prevalent in brain and soft tissue metastases among distant metastases.

**Conclusions:**

Our data strongly support an integrated analysis of the immune phenotype together with the PD-1/LAG-3 expression in distant metastases to identify patients with inflamed but exhausted tumors. This may eventually improve the stratification and likelihood for advanced breast cancer patients to profit from immunotherapy.

## Introduction

Immunotherapy with immune checkpoint inhibitors is usually considered in advanced metastatic breast cancer. The intention is to reduce the tumor burden by restoring a durable anti-tumor immune response. As breast cancer is not a highly immunogenic disease in general, treatment efficacy seems to depend on the molecular breast cancer subtype and the expression of PD-L1 [1, 2].

Monotherapy against programmed cell death protein-1 (PD-1) showed only the modest tumor and durable response rates in breast cancer (4–25%) [2]. In the need of novel strategies, in vivo studies provided convincing evidence that a dual blockade against PD-1 and the novel immune checkpoint receptor lymphocyte-associated gene-3 (LAG-3) [3] can result in tumor reduction and increase of survival [4, 5] by restoring CD8^+^ T cell function [6]. In human tumor tissue, LAG-3 is co-expressed with PD-1 on activated but exhausted CD8^+^ T cells [7]. Particularly, highly immunogenic tumors susceptible to immunotherapy like melanoma, microsatellite instable colorectal cancer, or triple-negative breast cancer carry PD-1^+^/LAG-3^+^ CD8^+^ tumor-infiltrating T cells [8].

In breast cancer, the predictive value of LAG-3 expression remains still unclear. There is some prognostic evidence associating high LAG-3 expression with improved overall survival (OS) but uncertain significance with respect to disease-free survival (DFS) [9]. Most ongoing clinical trials are investigating anti-LAG-3 drugs in combination with a dual blockade against PD-1 in solid tumors in advanced disease with promising survival benefits and long duration of response rates for those who profited [5].

Interestingly, most of these trials are considering neither the amount nor the distribution of tumor-infiltrating CD8^+^ T cells. For immunotherapy to be active though, CD8^+^ T cells must be present within the tumor bed—referred to as inflamed or clinically “hot” tumors [10–13]. Moreover, while the advanced metastatic disease is targeted, PD-1 and LAG-3 expression within distant metastatic breast cancer remains understudied. Their assessment within different anatomical sites of distant metastasis might however be crucial given that the composition of the tumor immune microenvironment is heterogeneous and is critically influenced by organ-specific parenchymal cells [14–16].

To address this knowledge gap, we assessed the CD8^+^ T cell immune phenotype as well as PD-1 and LAG-3 expression in primary tumors (PBTs) with intrapatient matched distant metastases (METs) in a retrospective cohort of 95 breast cancer patients by using immunohistochemistry on whole sections. Metastases had occurred at either brain, bone, liver, or soft tissue.

## Materials and methods

### Patient cohort

We searched breast cancer patients suffering from either invasive ductal or invasive lobular breast cancer with hematogenous metastases in the archives of the Department of Pathology and Molecular Pathology, University Hospital Zurich, in the time period of 1995–2019. We analyzed tissue material of 95 formalin-fixed paraffin-embedded (FFPE) PBTs and their corresponding sites of distant METs. Large parts of this cohort have been previously described [17]. Specifically, there were 49 surgical specimens and 46 biopsy specimens among the metastatic lesions. All PBTs had been characterized for estrogen (ER), progesterone (PR), and Her2 receptor expression either at the time of the primary diagnosis or retrospectively. To obtain a homogenous result for ER, PR, and Her2 receptor expression, all cases were re-classified according to the respective ASCO guidelines [18, 19]. Molecular breast cancer subtypes were defined by their ER, PR, and Her2 receptor expression and their Ki-67 proliferation rate according to the St. Gallen Consensus Conference [20, 21] as follows: luminal A (ER+ and/or PR+, HER2−, Ki-67 ≤ 14%), luminal B (ER+ and/or PR+, and/or HER2+, Ki-67 > 14%), triple-negative (ER−, PR−, HER2−), and Her2/neu (ER−, PR−, HER2+). Four patients were treated with preoperative chemotherapy, and the others underwent adjuvant treatment after surgery according to the time current guidelines and available regimens. Distant metastasis had occurred to either brain, bone, liver, or soft tissue with only one corresponding distant metastasis to each primary tumor (Table 1). Yet, we cannot exclude that patients suffered from additional metastatic sites not undergoing biopsy but being monitored by imaging only. In addition to this cohort of 95 patients, we included 43 additional breast cancer brain metastases without a corresponding primary tumor available (Table 2). Approval of the use of human primary breast cancer samples and metastatic tissue was obtained from the official ethical authorities of the Canton Zurich, Switzerland (ethical approval KEK-2012-553), according to the Declaration of Helsinki.

**Table 1:**
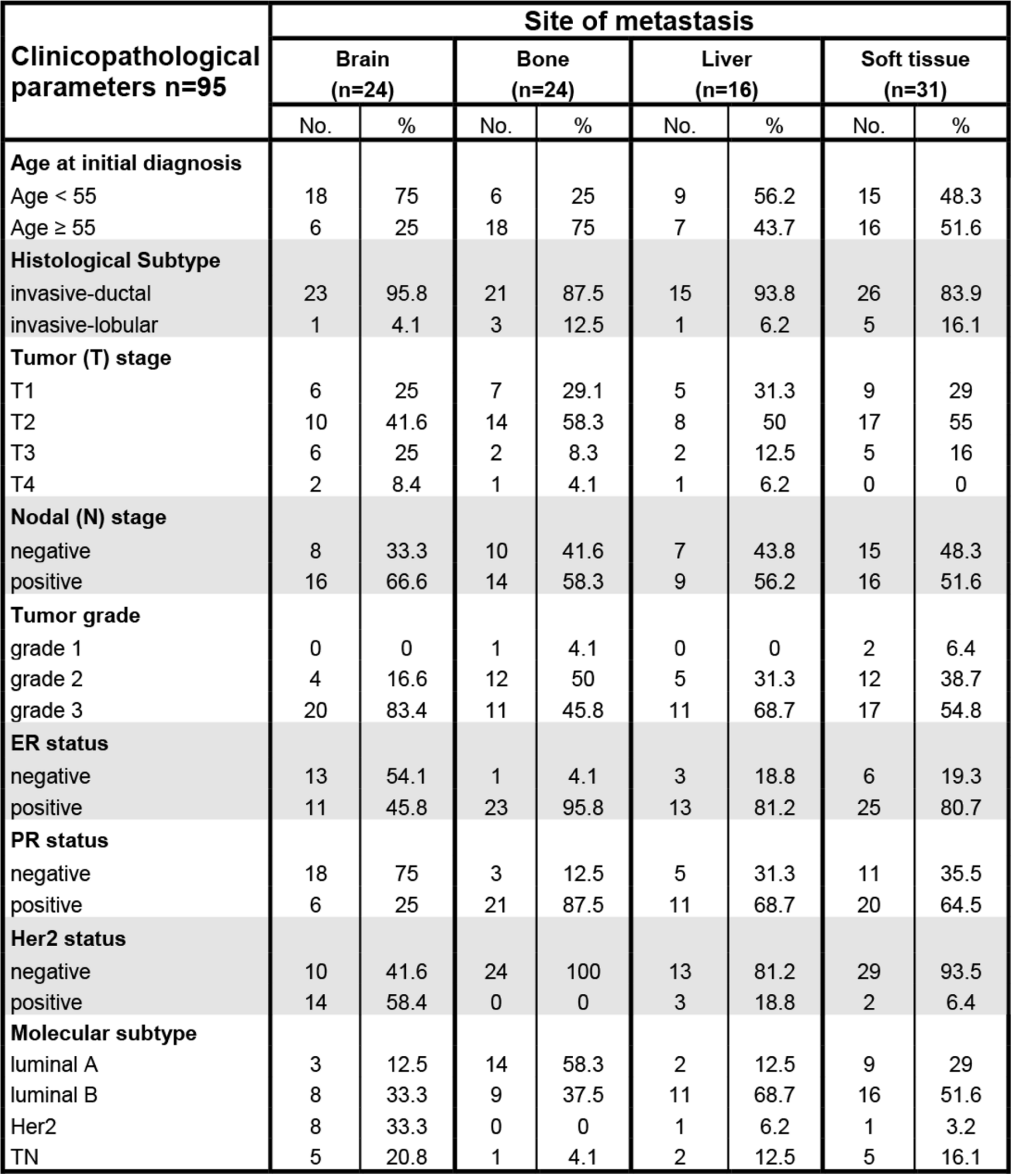
Clinicopathological data of the 95 breast cancer patients with metastasis at either of the four anatomical locations investigated

Clinicopathological data and group distribution of the 95 breast cancer patients with respect to the four anatomical locations at which the corresponding intrapatient metastasis had occurred. Percentages are shown in relation to the respective metastatic site

**Table 2:**
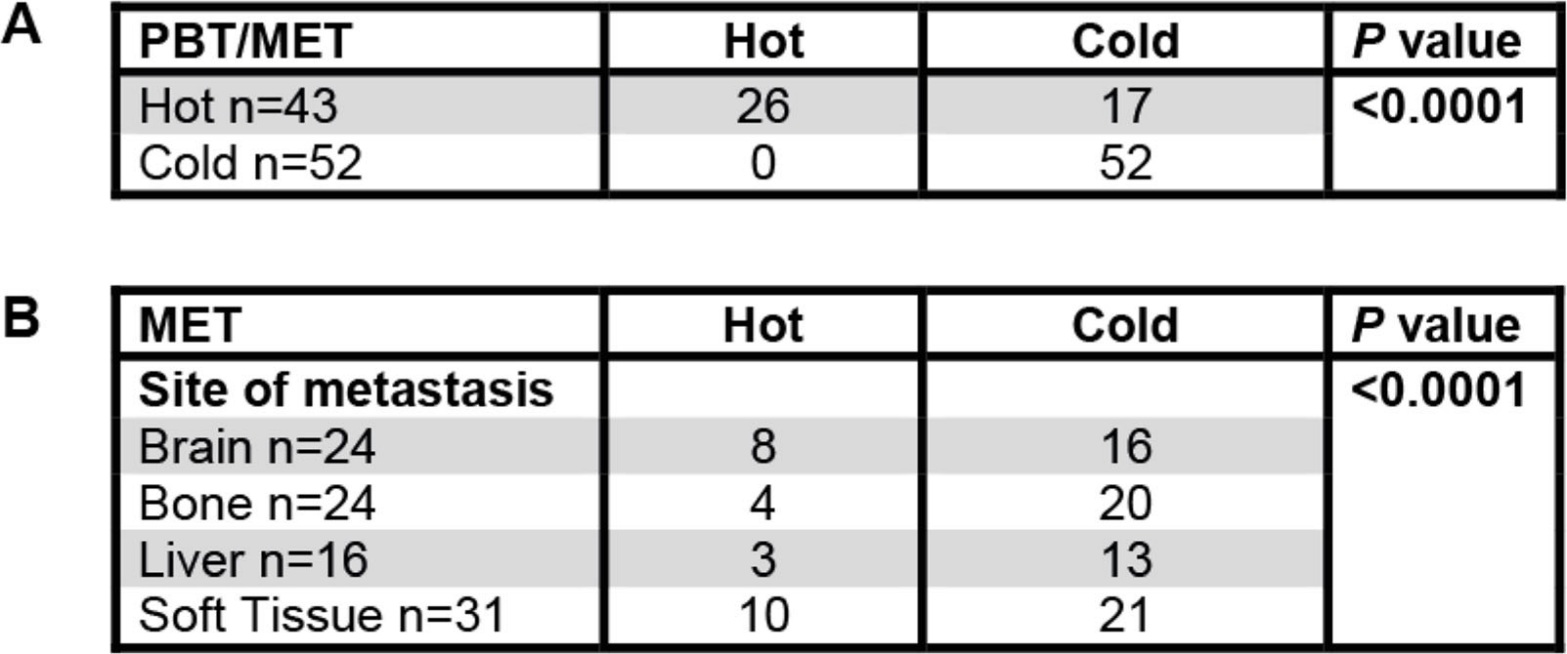
Distant METs of “cold” PBTs remain “cold”

PBTs (left column) with an inflamed/hot immune phenotype either stayed inflamed/hot or turned cold (excluded or desert) at their matched METs (top row). In contrast, cold/excluded PBTs (left column) always remained cold (excluded or desert) at their matched METs (top row) (A)

Among metastatic sites (left column), brain and soft tissue METs were more commonly hot/inflamed (top row) than liver and bone METs (B)

### Tissue selection and immunohistochemistry

In order to assess the CD8^+^ T cell immune phenotype together with PD-1/LAG-3 expression, three different tumor compartments must be available for analysis. We based our classification of a CD8^+^ T cell immune phenotype on their distribution within the three tumor compartments intratumoral, stromal, and invasive margin [18] defined as follows: intratumoral = intraepithelial compartment of the tumor consisting of tumor cells without intervening intratumoral stroma, stromal = intratumoral stroma without tumor cells, and invasive margin = the region centered on the border separating the host tissue from the malignant nests, with an extent of 1 mm. This is based on previous definitions [22] but refines the “central tumor” compartment into an intratumoral and stromal compartment. Therefore, we only studied large tissue sections of primary tumors and metastases. First, we screened each case by hematoxylin and eosin (HE) staining to assess lymphocytic infiltrate in the three tumor compartments. Mostly, lymphocytes were situated at the tumor margins. One tissue block per case was further analyzed by immunohistochemistry. Tissue blocks were cut in multiple 2-μm sections for immunohistochemical stainings. Immunohistochemical stainings of large tissue sections were performed using automated immunostainers (Ventana Medical Systems, Tucson, AZ, USA, or Leica BOND-III, Leica Microsystems, Heerbrugg, Switzerland) utilizing the antibodies monoclonal mouse anti-human CD8 (DAKO, clone C8/144B, dilution 1:100), monoclonal mouse anti-human lymphocyte activation gene 3 antibody (Abcam, clone 17B4, dilution 1:100), and monoclonal rabbit anti-human PD-1 antibody (Cell Signaling Technologies, clone D4W2J, dilution 1:100) with pretreatments according to the respective manufacturers’ instructions. Antibody detections were performed using Refine-HRP-Kit on BondMax Benchmark, Leica.

### Classification of the CD8^+^ T cell immune phenotypes

To translate the distribution pattern of tumor-infiltrating CD8^+^ T cells into an immune phenotype, we first scored their spatial distribution within the primary tumors and distant metastases according to the previously suggested consensus statement [22]. As we evaluated the whole slides capturing CD8^+^ T cell heterogeneity together with immunohistochemistry, we also evaluated the intratumoral/tumor epithelial compartment. We defined (i) immune desert if we could not find any CD8^+^ T cell in neither of the three tumor compartments, (ii) immune excluded if CD8^+^ T cells had arrived at the tumor environment but could only be found at the invasive margin or within the stromal but not within the intratumoral compartment, and (iii) inflamed if CD8^+^ T cells could be detected in the stromal compartment but, importantly, also in direct contact with tumor cells meaning that they had properly infiltrated the intratumoral compartment essential for a CD8^+^ T cell-mediated cytotoxicity. Due to intratumoral heterogeneity, we evaluated at least three different tumor areas per slide and considered the most common pattern as the predominant immune phenotype. Immune phenotypes were assessed in the first run by two pathologists (ZV, BS) and were randomly re-assessed by one investigator (BS) after a period of at least a few weeks.

### Scoring of LAG-3 and PD-1

Immunohistochemistry of LAG-3 presented a dot-like membranous staining (Fig. 2a) as also depicted by the manufacturer and as recently described [23]. Scoring of LAG-3 and PD-1 was performed as described recently [23]. Any membranous positive expression of the former within the three tumor compartments was regarded to be sufficient to dichotomize the particular compartment into positive or negative. To minimize bias due to tumor heterogeneity, we evaluated three different areas per tumor compartment of each tumor section. The average results of these three fields per tumor compartment were used for further evaluation. Scores were assessed in the first run by two pathologists (ZV, BS) and were randomly re-assessed by one investigator (BS) after a period of at least a few weeks.

### Statistical analysis

Associations between immune phenotypes with respect PBTs, METs, and PD-1/LAG-3 expression were performed by the Fisher-Freeman-Halton test. To this end, immune desert and excluded tumors were summarized into “cold” tumors and inflamed tumors were categorized as “hot.” Disease-free survival (DFS) was defined as the time between diagnosis of the PBT and the occurrence of the respective distant MET. While this is in contrast to the common perception of DFS in clinical trials, we are convinced that this time interval may serve as an adequate marker in the here presented exploratory setting. Survival analysis was computed using the Kaplan-Meier estimator. To compare the Kaplan-Meier survival estimates, the log-rank test was applied as statistical analysis. Significances are displayed as follows: ns = *p* > 0.05, *p ≤ 0.05, **p ≤ 0.01, ***p ≤ 0.001, and ****p ≤ 0.0001. Statistical analysis was performed using the GraphPad Prism (version 7.04) and StatXact (version 12; Cytel Studio) software.

## Results

### Distant METs of “cold” PBTs remain “cold”

**Fig. 1:**
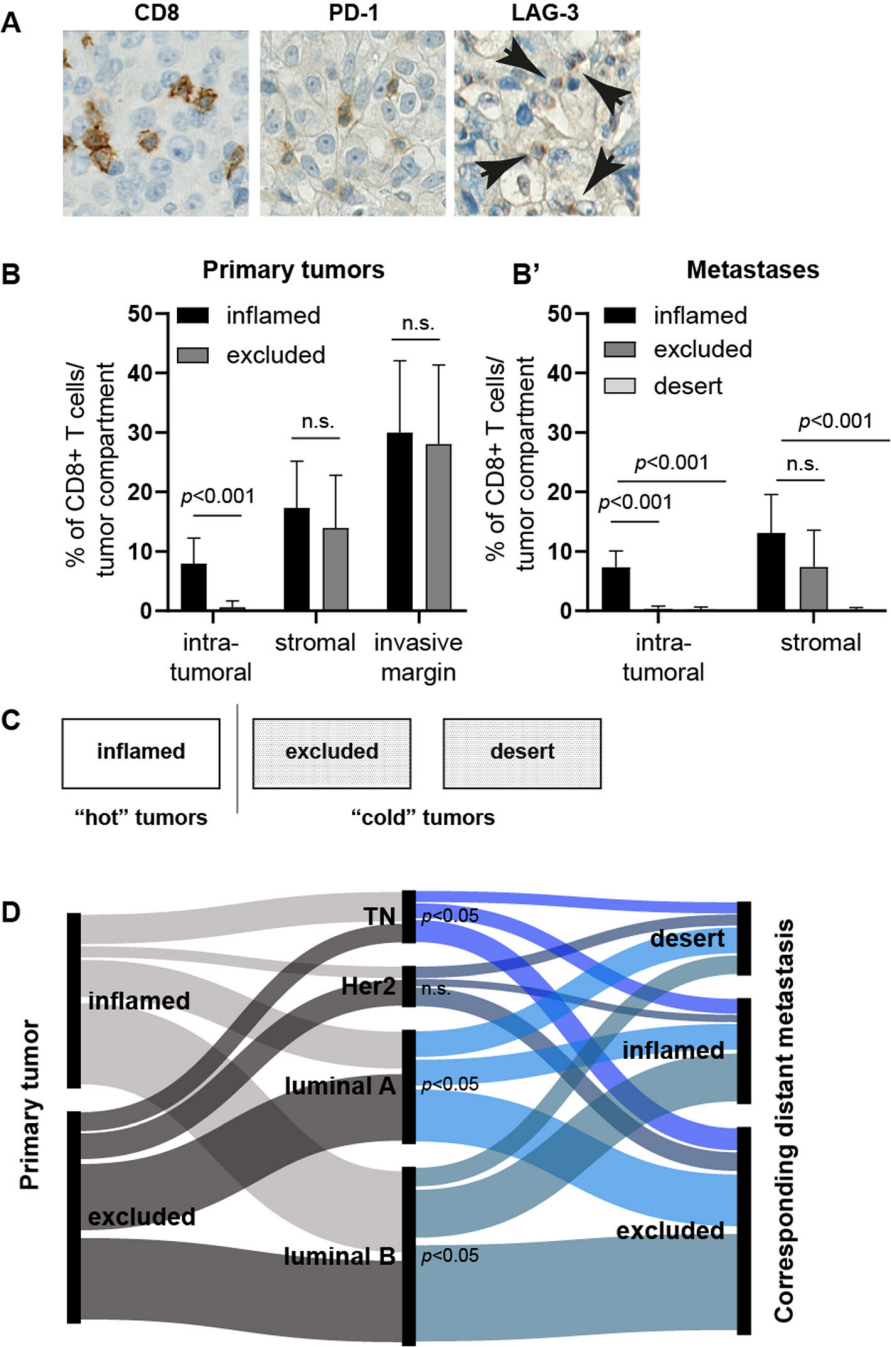
Immune phenotype turns “cold” in matched distant metastases irrespective of molecular subtype. Whole breast cancer tissue sections were stained for CD8, PD-1, and LAG-3 (**a**). CD8^+^ T cells were scored semiquantitatively and assigned to the proposed immune phenotype (**b**). Based on the CD8^+^ T cell distribution pattern within the tumor compartments, the samples were classified into “hot”/inflamed or “cold”/excluded/desert tumors (**c**). An alluvial plot [24] was used to depict the distribution of immune phenotypes within the primary tumor (left) and the corresponding distant metastases (right) among the molecular breast cancer subtypes (middle). Metastases turned into “cold” tumors irrespective of the molecular subtype (**d**)

We performed immunohistochemistry for CD8, PD-1, and LAG-3 (Fig. 1a) on whole tissue sections. To translate the frequency and spatial distribution of CD8^+^ T cells within the tumor compartments to a certain immune phenotype, we first semiquantitatively evaluated CD8^+^ T cells within PBTs and METs (Fig. 1b) to categorize them into three immune phenotypes corresponding to the clinical categories “hot” and “cold” (Fig. 1c). In PBTs, the frequencies of immune excluded and inflamed cases were similar. In contrast, METs turned “cold” regardless of the breast cancer molecular subtype as depicted using an alluvial diagram (Fig. 1d), clinicopathological parameters, or the anatomical site of the METs (data not shown). Interestingly, METs of “cold” PBTs always remained “cold” at their matched metastatic site. In contrast, “hot” PBTs either stayed “hot” or turned “cold” in their corresponding METs (Table 2 (A)). Among distant metastatic sites, brain and soft tissue METs remained more commonly inflamed (Table 2 (B)).

### PD-1^+^/LAG-3^+^ expression correlates with a “hot” immune phenotype

LAG-3 expression occurred only in PD-1-positive cases (*p* < 0.01; not shown) with an overall low frequency (5% positive intratumoral and 31% positive stromal cases in PBTs with lower frequencies for METs). PD-1^+^/LAG-3^+^ expression correlated significantly with a “hot” immune phenotype in both PBTs and METs (Table 3 (A and B)) regardless of the molecular breast cancer subtype (not shown). Brain and soft tissue METs displayed more commonly PD-1 expression with the same tendency for LAG-3 (Table 3 (C)).

**Table 3:**
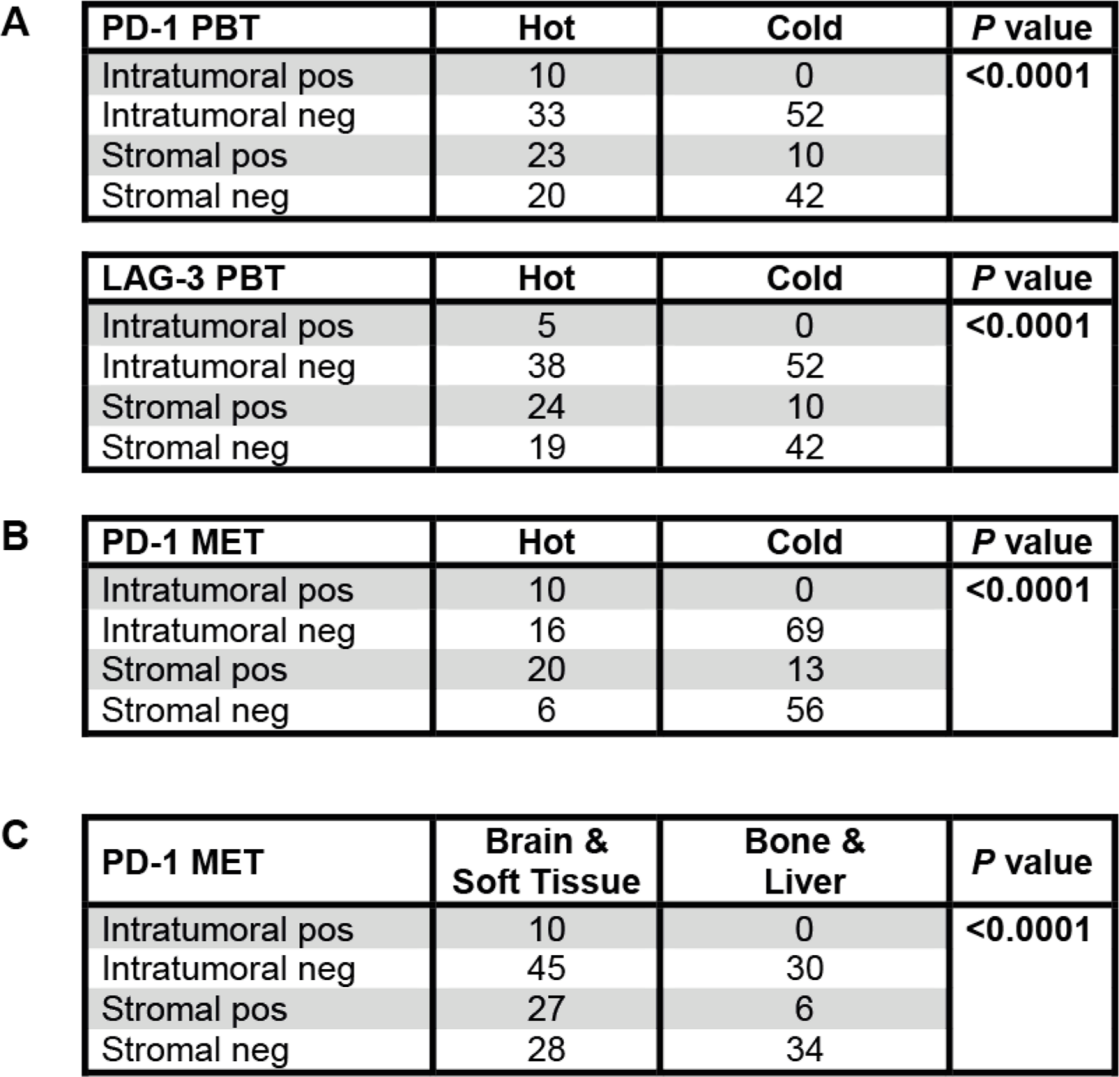
Immune phenotype correlates with PD-1 and LAG-3 expression

PD-1 and LAG-3 expression correlated both in the PBTs (A) and the METs (B) with a hot/inflamed immune phenotype. PD-1 intratumoral positivity was most predominantly overserved in brain and soft tissue METs (C)

### Negative prognostic disease-free survival impact of PD-1 and LAG-3 expression

**Fig. 2:**
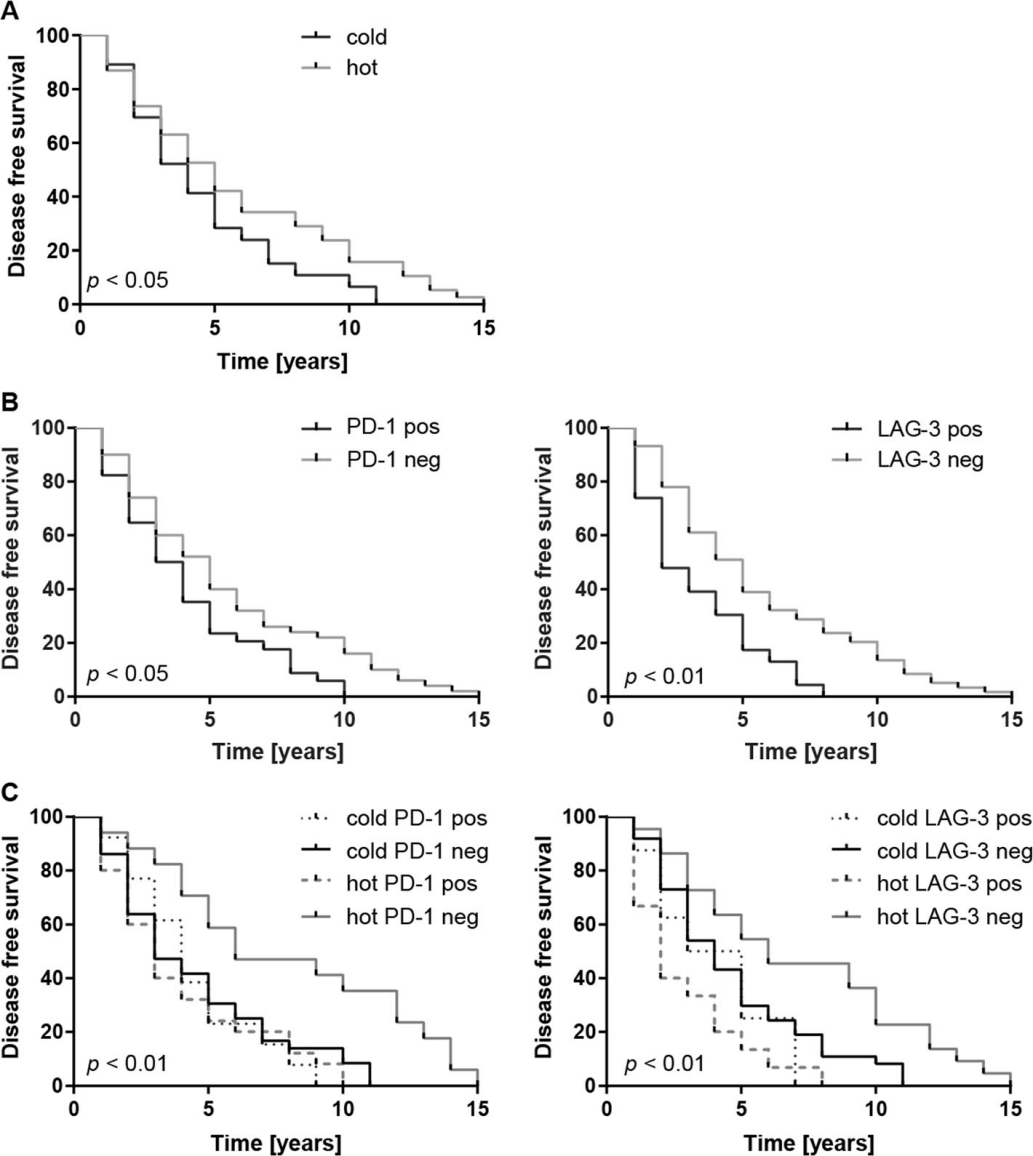
Negative prognostic disease-free survival impact of PD-1 and LAG-3 expression. While a “hot” immune phenotype correlated with improved DFS (**a**), stromal positivity for either PD-1 or LAG-3 was associated with a worse DFS (**b**). The combined analysis of the immune phenotype and PD-1/LAG-3 expression further stratified the DFS showing that “hot” but PD1^−^/LAG-3^−^ cases displayed the best DFS (**c**)

The dichotomization of PBTs into inflamed/“hot” or excluded-desert/“cold” tumors revealed an improved disease-free survival (DFS) for “hot” PBTs (Fig. 2a). The combined analysis of PD-1/LAG-3 expression within the stromal tumor compartment and immune phenotype further improved DFS discrimination: “hot” but PD-1^−^ PBTs displayed a significantly better DFS, while “hot” but PD-1^+^ PBTs showed the same reduced DFS as “cold” PD-1^+^ or PD-1^−^ tumors (Fig. 2b, c).

### PD-1^+^/LAG-3^+^ expression is associated with “hot” brain metastases

Among distant metastatic sites, brain and soft tissues showed more prevalently an inflamed, PD-1^+^/LAG-3^+^ immune phenotype. Due to our cohort, it remained unclear whether this effect was due to the molecular breast cancer subtype or the brain/soft tissue-specific tumor microenvironment. As the brain is commonly regarded as an immune-privileged organ and brain metastases are clinically highly relevant, we included additional 43 breast cancer brain metastases (Table 4) resulting in 67 brain metastases in total. About half of these additional brain METs displayed a “hot” immune phenotype. Again, PD-1^+^/LAG-3^+^ expression correlated with an inflamed immune phenotype (Table 5) but was not associated with the molecular breast cancer subtype (not shown).

**Table 4:**
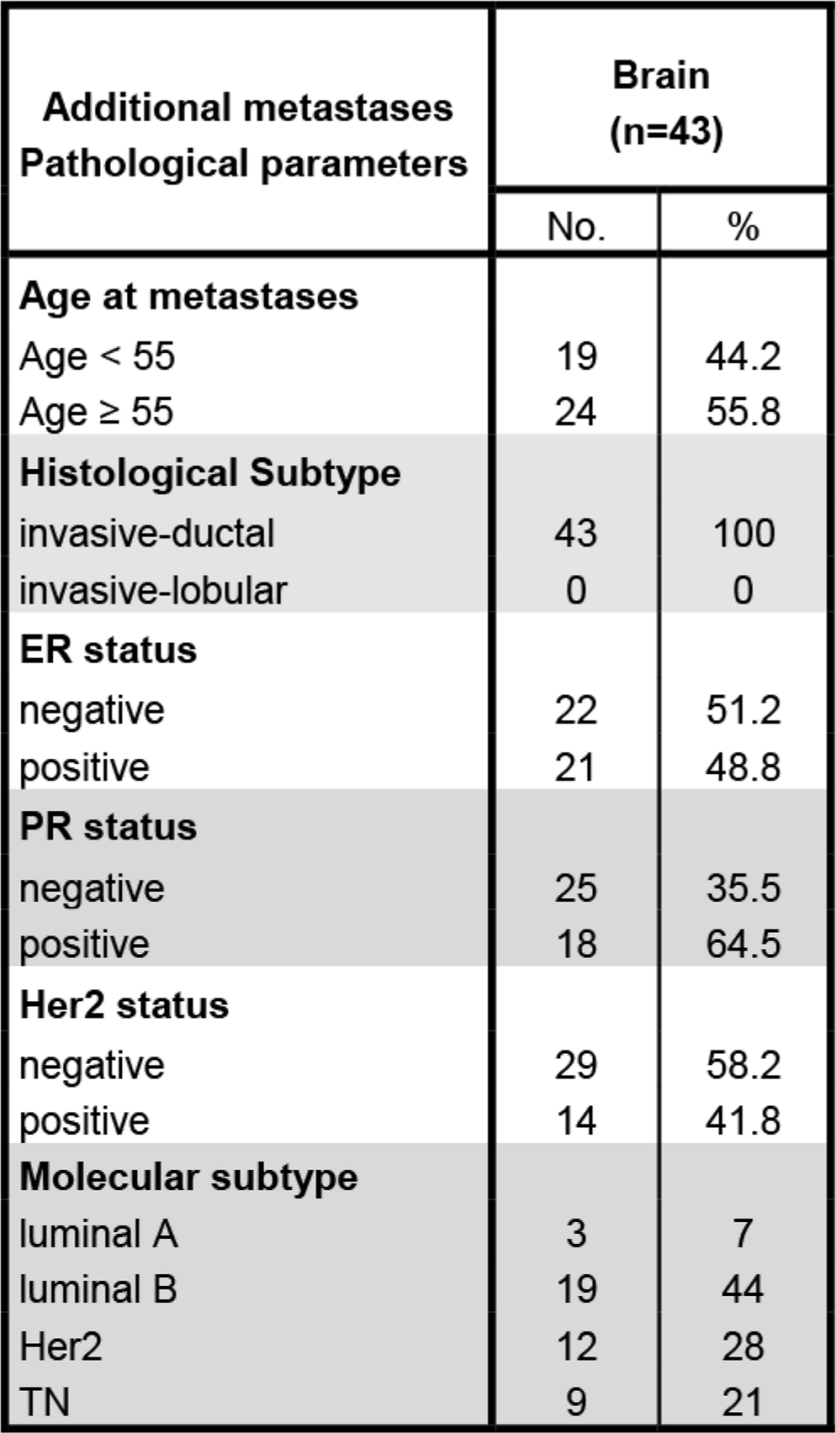
Clinicopathological parameters of the additional 43 brain metastases

Overview of the additional cohort of breast cancer brain metastases. The primary breast cancer was not available in these cases

**Table 5:**
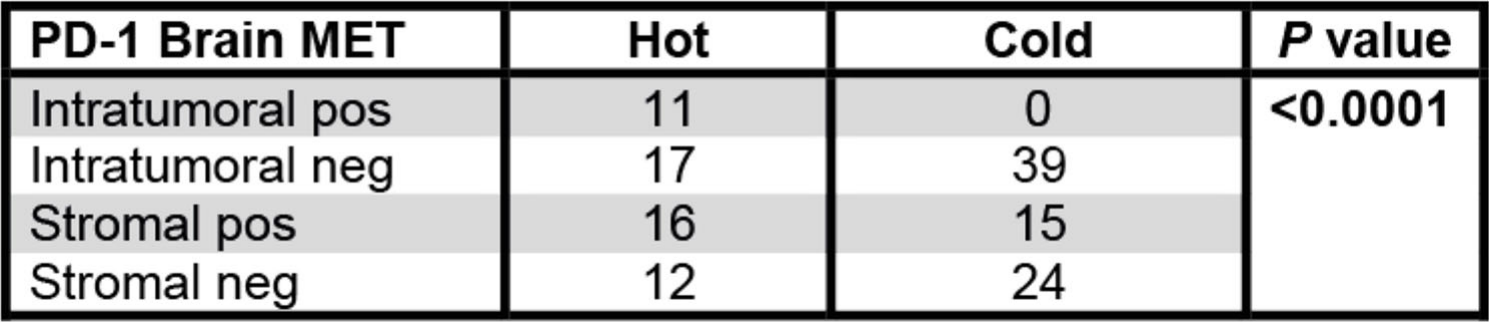
PD-1 expression correlates to an inflamed phenotype in all investigated brain metastases

In the enlarged brain metastasis cohort comprising additional 43 breast cancer brain metastases to the initial 24 brain metastasis PD-1 expression also correlated to a hot/inflamed immune phenotype

## Discussion

By investigating a large intrapatient matched PBT distant MET breast cancer cohort [2, 17, 25–27], we have shown that (i) PD-1^+^/LAG-3^+^ is strongly associated with a “hot” immune phenotype and (ii) differs between METs and PBTs. As described, LAG-3 expression was only observed in PD-1-positive cases [7] with an overall low frequency [23].

For this study, we translated the intratumoral spatial distribution pattern of CD8^+^ T cells into three defined immune phenotypes, intended to reflect the clinical terms “hot” or “cold” tumors. We based our evaluation on the following tumor compartments: tumor center comprising the intratumoral/tumor epithelial and stromal compartment and the invasive margin compartment. While these compartments are arbitrary to a certain extent since T cells are thought to freely move through tissue and are as such not stuck to a particular compartment as implied by a snap-shot-like impression in FFPE tissue, these three compartments are well-established and proposed as such by consensus agreements [22].

As published previously [17], we evaluated the intratumoral compartment of the “tumor center” [22] compartment based on the notion that a direct contact between CD8^+^ T cells and tumor cells must occur for CD8^+^ T cell-mediated cytotoxicity. Within this concept, also metastatic biopsies containing the “tumor center” fulfill the necessary criteria to identify immune phenotypes as the value of the invasive margin—often lacking in metastatic biopsy material—becomes limited. Systematic studies assigning the spatial distribution of CD8^+^ T cells to a certain immune phenotype and, moreover, validating the biological significance of these immune phenotypes to the response to immune checkpoint inhibition are lacking up to date. There is thus no consensus classification of immune phenotypes yet. While we are convinced that our proposed immune phenotype classification may serve as a valuable surrogate marker also applicable in metastatic tissue, our suggested immune phenotypes certainly need to be validated in a cohort that includes responders and non-responders to immune checkpoint inhibition.

In contrast to the previous results, neither the immune phenotype nor PD-1/LAG-3 expression was associated to the molecular breast cancer subtype [28] possibly due to the overall small number of patients in our cohort.

Overall, METs turned “cold” suggesting reduced immunogenicity of METs in general [25]. Interestingly, METs of “cold” PBTs always remained “cold” at their matched metastatic site, while “hot” PBTs either stayed “hot” or turned “cold” in their corresponding METs. These observations were independent of the breast cancer molecular subtype. While the underlying mechanisms remain unclear, our findings imply a tumor intrinsic immunogenicity and may explain the low response rates to immunotherapy in metastatic breast cancer [2, 5].

From a clinical translational point of view, these results strongly favor the spatial assessment of CD8^+^ T cells together with PD-1/LAG-3 within metastatic tissue if immune modulatory therapy is considered. In case of synchronous metastases at different anatomical locations, biopsy material of either all metastatic sites or one of clinically greatest importance, such as brain metastases, should be discussed. The value of a combined assessment of PD-L1 and tumor-infiltrating lymphocytes was recently proposed as a more comprehensive immuno-oncological biomarker in breast cancer [29]. Whether our suggested evaluation of immune phenotypes together with PD-1/LAG-3 within metastatic tissue may serve as an even more comprehensive immuno-oncological biomarker needs further validation in larger and prospective cohorts.

Among the distant metastatic sites, brain and soft tissue METs displayed more prevalently an inflamed but exhausted immune phenotype. To distinguish site-specific immune changes [14] from the molecular breast cancer subtype, we included additional brain metastases. Again PD-1^+^/LAG-3^+^ expression correlated to “hot” brain METs regardless of the molecular breast cancer subtype supporting our previous notion of a tumor intrinsic immunogenicity.

In our cohort, an inflamed PD-1^−^/LAG-3^−^ immune phenotype in the PBT was associated with an improved DFS implying a negative DFS prognostic impact of PD-1/LAG-3 expression. While this certainly needs to be confirmed in a larger cohort, this adverse prognostic significance of PD-1/LAG-3 expression was not unexpected given their inhibitory effects on the immune response in general [6]. Nevertheless, these observations were in contrast to a recent publication describing improved DFS in PD-1- and LAG-3-positive primary breast cancers [23].

In recent reports, LAG-3 expression is associated with different DFS prognostic outcomes which may be due to a small number of LAG-3/PD-1 positive cases, heterogenous methods employed, and different LAG-3-positive cutoffs [9]. While Burugu et al. [23] used tissue microarrays (TMAs) and focused on intratumoral lymphocytes, the paper by Bottai et al. [28] described data using whole slides and stromal lymphocytes. This illustrates the inconsistencies and limitations between methods and the tumor compartment evaluated. TMAs are usually constructed using only small tumor cores taken in regions with high tumor content and not selected based on the presence of abundant immune infiltration. TMAs may as such neither reflect the intratumoral heterogeneity nor give the complete picture of the presence of LAG-3-positive cells as the whole tissue sections we used. Furthermore, due to our interest in LAG-3 and PD-1 expression in metastatic tissue, our cohort is biased for patients with advanced metastatic disease thus differing from an average breast cancer cohort.

Our study fills an important knowledge gap in metastatic breast cancer in two main regards: (i) the immune phenotype and PD-1/LAG-3 expression within metastatic breast cancer are significantly different from the primary tumor and among anatomical metastatic sites and (ii) PD-1^+^/LAG-3^+^ expression is strongly associated with a “hot” immune phenotype. Taken together, not the primary tumor but metastatic breast cancer should be analyzed for the immune phenotype and PD-1/LAG-3 expression to reveal the metastasis-associated immune pathology. This dual evaluation in metastatic sites may eventually improve the stratification of advanced breast cancer patients for immunotherapy given that CD8^+^ T cells must be present within the tumor bed for an effective immunotherapy response [10].

## Conclusions

In summary, LAG-3 was exclusively observed in PD-1^+^ cases with an overall low frequency. PD-1^+^/LAG-3^+^ expression was associated with a “hot” immune phenotype both in PBTs and METs regardless of the breast cancer molecular subtype. Disease-free survival was significantly improved in inflamed but PD-1^−^/LAG-3^−^ PBTs. In our cohort, METs of “cold” PBTs always remained “cold” at their matched metastatic site. In contrast, “hot” PBTs either remained “hot” or turned “cold” in their corresponding METs. Among the anatomical sites of metastases, brain and soft tissue metastases were more commonly inflamed with signs of exhaustion.

Our study emphasizes the careful assessment of the immune phenotype and PD-1/LAG-3 expression in metastatic breast cancer tissue to overcome intrapatient tumor heterogeneity. Furthermore, analysis of metastatic breast cancer tissue may improve the stratification of advanced breast cancer patients for a dual anti-PD-1/anti-LAG-3 immunotherapy.

## Data Availability

The datasets used and/or analyzed during the current study are available from the corresponding author on reasonable request.

## Abbreviations

PBT: Primary breast tumor
MET: Distant metastasis
PD-1: Programmed cell death receptor 1
LAG-3: Lymphocyte activation gene 3
FFPE: Formalin-fixed paraffin-embedded
DFS: Disease-free survival
HE: Hematoxylin and eosin
TMA: Tissue microarray
